# A single-arm, open-label pilot study of neuroimaging, behavioral, and peripheral inflammatory correlates of mindfulness-based stress reduction in multiple sclerosis

**DOI:** 10.1038/s41598-024-62960-w

**Published:** 2024-06-18

**Authors:** Christopher C. Hemond, Mugdha Deshpande, Idanis Berrios-Morales, Shaokuan Zheng, Jerrold S. Meyer, George M. Slavich, Steven W. Cole

**Affiliations:** 1 Department of Neurology, University of Massachusetts Chan Medical School, 55 Lake Avenue North, Worcester, MA 01655 USA; 2 Department of Radiology, University of Massachusetts Chan Medical School, Worcester, MA 01655 USA; 3 Department of Psychological & Brain Sciences, University of Massachusetts Amherst, Amherst, MA 01003 USA; 4 Department of Psychiatry and Biobehavioral Sciences, University of California, Los Angeles, CA 90095 USA

**Keywords:** Mindfulness-based stress reduction, Conserved transcriptional response to adversity, Hair cortisol, Patient reported outcomes, Perceived stress, Multiple sclerosis, Neuroimmunology, Chronic inflammation, Stress and resilience, Neurological disorders, Magnetic resonance imaging

## Abstract

Multiple sclerosis (MS) is a chronic neurological disease frequently associated with significant fatigue, anxiety, depression, and stress. These symptoms are difficult to treat, and prominently contribute to the decreases in quality of life observed with MS. The underlying mechanisms of these “silent” symptoms are not well understood and include not just the psychological responses to a chronic disease, but also biological contributions from bidirectional psycho-neuro-immune (dys)regulation of systemic inflammatory biology. To address these issues, we conducted a prospective, observational pilot study to investigate the psychological, biological, and neuroarchitecture changes associated with a mindfulness-based stress reduction (MBSR) program in MS. The overarching hypothesis was that MBSR modulates systemic and central nervous system inflammation via top-down neurocognitive control over forebrain limbic areas responsible for the neurobiological stress response. 23 patients were enrolled in MBSR and assessed pre/post-program with structural 3 T MRI, behavioral measures, hair cortisol, and blood measures of peripheral inflammation, as indexed by the Conserved Transcriptional Response to Adversity (CTRA) profile. MBSR was associated with improvements across a variety of behavioral outcomes, as well as on-study enlargement of the head of the right hippocampus. The CTRA analyses revealed that greater inflammatory gene expression was related to worse patient-reported anxiety, depression, stress, and loneliness, in addition to lower eudaimonic well-being. Hair cortisol did not significantly change from pre- to post-MBSR. These results support the use of MBSR in MS and elucidate inflammatory mechanisms related to key patient-reported outcomes in this population.

## Introduction

Multiple sclerosis (MS) is an inflammatory and neurodegenerative disease characterized by immune-mediated demyelination of the central nervous system^1^. One trigger for an inflammatory flare is psychological stress, as demonstrated through prospective MRI trials and retrospective cohort and case-control studies^2–4^. The effect size of stress on risk of disease flare is moderate (d = 0.53), and clinically relevant, exceeding the effect size for first-generation MS therapeutics like interferon-beta and glatiramer acetate (decreased risk, d ~ 0.3)^2^. A recent randomized controlled trial showed that a cognitive-based stress reduction intervention temporarily reduced the number of new inflammatory brain lesions in MS by over 50%, acting synergistically with MS medication^5,6^.

Many MS patients experience high rates of stress, depression, fatigue, anxiety, and cognitive dysfunction, the so-called “silent symptoms” that are not reflected in typical neurological disability grading scales. These “silent” symptoms are highly prevalent, underrecognized, disabling, and represent an unmet care need in MS. Most if not all FDA-approved disease modifying therapies (DMT) do little to improve these symptoms; for example, first-line DMTs only modestly improve^7^, or decrease^8^ quality of life measures in MS patients. A recent survey showed that 100% of MS patients rely on at least one complementary or integrative health intervention to address unmet symptomatic needs^9^.

Mindfulness-Based Stress Reduction (MBSR) may help address some of these unmet needs and offer a solution to common problems in stress-reduction services that are typically difficult to access and scale (most require one-on-one interactions), prohibitively costly, nonstandardized, and can have high attrition rates. MBSR was initially developed at the University of Massachusetts Medical School by Professor Jon Kabat-Zinn, and is a manualized group intervention that teaches a set of tools to cultivate present moment awareness with an attitude of non-judgement and acceptance^10^. MBSR has salubrious psychological effects on both clinical and healthy populations. A meta-analysis of MBSR training in 2668 healthy individuals showed moderate improvements in perceived stress, anxiety, depression, distress, and quality of life, with larger effect sizes for the standardized 8-week course compared to shortened alternatives^11^. Populations with higher baseline stress seemed to benefit more^11,12^. Moreover, a study in persons with MS by Grossman and colleagues found durable improvements in quality of life, depression, and fatigue at 6-month follow-up compared to an educational control group^13^.

The neurobiological mechanisms of MBSR’s efficacy remain incompletely understood. One potential mechanism is via the psycho-neuro-immune axis, such that reducing stress would mitigate the pro-inflammatory potential of the innate immune system through top-down efferent pathways involving the sympathetic nervous system (SNS) and hypothalamic–pituitary–adrenal (HPA) axis^14^. This inflammatory response to stress can be measured as the “conserved transcriptional response to adversity” (CTRA)^15,16^, a gene expression profile first observed in persons with chronic stress and loneliness^17^. The CTRA is characterized by upregulation of inflammatory gene expression, and downregulation of genes related to antibody synthesis and innate antiviral response^18^, factors which remain intriguing and relevant given the strong evidence for Epstein-Barr Virus as a cause of MS^19,20^. Early data have shown that MBSR may produce anti-inflammatory effects^21–23^, beneficial changes to neuroendocrine stress hormone release^24^, and changes in structural neuroarchitecture relevant to stress^25–27^.

There is considerable convergence between neurological substrates of MBSR, stress, and the sympathetic autonomic regulatory network that has been shown to modulate the CTRA. Most of the shared anatomy is related to limbic and paralimbic structures such as the hippocampus, amygdala, cingulate cortex, prefrontal cortex and insula^28–30^. Some^25,27^ (but not all^31^) studies have shown structural changes in limbic areas following MBSR; few studies have explored how the CTRA changes in relation to formal MBSR^23^ or its similar mindful aware practices^32–36^. No studies to our knowledge have assessed the structural neural correlates of cross-sectional or longitudinal changes in the CTRA.

## Present study

To explore these ideas, we conducted a longitudinal, observational, unblinded, single-arm study of persons with MS (pwMS) who had been recommended (or chosen) to participate in an MBSR course. We collected pre-and post-MBSR patient-reported outcome measures, hair cortisol, MRI scans, and blood samples that were assessed for the CTRA profile. Based on the literature reviewed above, we hypothesized that participating in MBSR would be associated with group-level improvements in self-reported stress and anxiety measures, and, additionally, that these measures would relate to biological changes as measured by serum inflammatory markers, reduced long-term cortisol output (as measured in the hair), and MRI volumetric changes at the patient-level. Specifically, we hypothesized that structural changes in a set of cerebral gray matter structures involved in central (cerebral) autonomic control would predict improvements in peripheral inflammatory measures as assessed by the CTRA. We chose 13 (lateralized) apriori limbic/paralimbic regions of interest (ROI) to analyze based on overlapping literature between (1) structures previously shown to be associated with MSBR and (2) structures with potential modulatory influence on the hypothalamic-pituitary-axis (HPA) and sympathetic (autonomic) output. We chose ROIs including the amygdala, hippocampus, hypothalamus, brainstem, insula, anterior cingulate, and subcallosal structures, all of which could plausibly affect peripheral immune functioning via top-down modulation of the “master regulatory” structures of neuroendocrine and autonomic efferent pathways, including the paraventricular nucleus of the hypothalamus^37^ and other brainstem nuclei^38^. We did not assess other brain regions as we anticipated limited power for the study and were concerned about type I (false positive) statistical errors. Assessing longitudinal patient self-reported outcome metrics in parallel with biological outcomes is fertile territory for exploring which self-reported outcomes may carry more biological influence, and therefore are most important to target from a clinical perspective.

## Method

### Study design and participants

This is a prospective, pre/post observational cohort study of pwMS who chose to participate in a MBSR program at the University of Massachusetts. Patients were referred to MBSR classes either by their healthcare provider or contacted the research staff via poster advertisements in the clinic. Patients in this MS clinic are able to enroll in MBSR classes free-of-charge through a separate grant, and participating in this research was entirely voluntary. Participants were not paid. Inclusion criteria were: age 18–75, and having a diagnosis of multiple sclerosis without any clinical exacerbation in the prior 6 months. Exclusion criteria included those who had taken MBSR or were enrolled in dedicated mindfulness training in the prior 10 years, severe psychiatric comorbidities (schizoform spectrum), or persons on nonselective beta-blockers (starting in January 2020, due to potential disruption of inflammatory gene expression outcomes). We did not exclude patients taking stable doses of serotonergic/noradrenergic reuptake inhibitors, tricyclic antidepressants, neuropathic pain medications, anti-spasticity agents, or anti-fatigue medications. No patients reported taking illicit substances.

A baseline research visit occurred between 1 and 21 days prior to the introductory session of the MBSR course. At this visit, patients underwent hair sample collection, a 3T MRI scan, and (starting January 2020) venipuncture. Questionnaires for self-reported outcomes were completed either on paper at the time of the visit, or any time prior to the start of the course using the online platform RedCap. Follow-up clinic visits were arranged to occur within 3 weeks of course completion. Participants undergoing MBSR at the start of the COVID-19 pandemic incurred a delay or cancellation of their follow-up visit due to temporary shutdown of research facilities. Patients were enrolled between April 2019 and September 2022.

106 pwMS were referred/interested in the mindfulness course (9 men, 97 women); 33 ultimately enrolled. Of these 33 patients, 23 (70%) chose to participate in the observational research and were included in this analysis. 96% (22/23) completed the MBSR course. The mean age ± SD of the cohort at baseline was 45.6 ± 11.3 years; all patients were female and classified as relapsing–remitting MS phenotype (Table 1). No adverse events were reported or noted.

**Table 1:** Cohort summary.

	N = 23
Age (years)	45.6 ± 11.3
Disability score (EDSS)	1.5 (1.0, 6.0)
Symbol digit modalities test	60 (42, 77)
Disease duration (years)	13.4 ± 7.7
Sex	
Female	23 (100%)
Disease modifying therapy	
Alemtuzumab	1 (4.3%)
B-cell depletion	13 (57%)
Dimethyl fumarate	3 (13%)
Glatiramer acetate	1 (4.3%)
Interferon beta-1a	1 (4.3%)
None	4 (17%)

Mean ± SD; Median (Range); n (%).

None of the participants experienced a clinical MS flare on-study, nor was there any evidence of on-study inflammatory disease activity based on stability of T2-hyperintense lesion number and volumes on pre- and post-MRI scans (data not shown). No patient changed disease modifying therapy over the course of the study.

### Class description

The MBSR course at UMass is a secular, manualized protocol consisting of weekly, 2.5-h interactive didactic and practicum sessions with the goal of learning non-judgmental present moment awareness of emotions and thoughts. There is a component of gentle standing yoga as well, which can be optionally performed in a chair. The class duration is 8 weeks, with an additional 8-h “all-day” session typically occurring during weeks 5 or 6. This class was initially based in-person, prior to the onset of the COVID-19 pandemic (March 2020), after which it was permanently switched to a virtual format (September 2020). All teachers were MBSR-certified.

### Patient reported outcomes

Pre- and post-MSBR psychosocial questionnaire assessments included the Brief Inventory of Perceived Stress^39^; Depression, Anxiety, and Stress Scale (DASS-21)^40^; UCLA Loneliness scale^41^; Modified Fatigue Impact Scale (5-item)^42^; and other measures that were administered but not the focus of the present analysis.

### Clinical data

Patient clinical and demographic data were obtained with patient consent through a clinical query of their electronic medical record. This included their neurological disability scores (the Expanded Disability Status Scale^43^, ranging from 0 = no objective disability to 10 = death from MS), cognitive processing speed (the symbol digit modalities test), disease duration, and treatment records including medications and use of disease-modifying therapies. Any use of glucocorticoids were recorded and converted to prednisone-equivalent dosing; this exposure was not uncommon given the frequent use of B-cell depleting agents requiring glucocorticoids as a premedication. The patient cohort exhibited overall low neurological disability (EDSS median = 1.5, ranging between 1.0 and 6.0), and none were below the threshold on the symbol digit modalities test (< 40) concerning for cognitive impairment (Table 1)^44^.

### Hair cortisol

Hair samples were collected containing approximately 50–100 individual hairs, cutting as close as possible to the surface of the scalp in the area of the vertex. Samples were immediately trimmed to a length of 2.5 cm as measured from the base of the follicle and sealed in aluminum foil pouches. Samples were stored at room temperature for less than 4 weeks before being shipped in batches to the University of Massachusetts Amherst for later processing and analysis. Hair samples were processed and analyzed according to previously described methods^45^ with minor modifications. Briefly, each sample was weighed, washed twice with isopropanol to remove external contaminants, and then ground to a fine powder. The samples were then extracted overnight in methanol, the methanol was evaporated followed by reconstitution of the extract in assay buffer, and the reconstituted extract was then spin-filtered to remove any residual solid material. Lastly, cortisol was analyzed in duplicate along with standards and quality controls using the Arbor Assays DetectX enzyme immunoassay. Intra- and inter-assay coefficients of variation are both < 10% for this assay.

### Gene expression analysis

Blood samples were collected into PAXgene RNA tubes according to manufacturer instructions, and stored upright at − 80 °C. Samples were sent and processed in a single batch at the UCLA Social Genomics Core. The CTRA was assessed as has been described previously^46–48^. Briefly, total RNA was extracted from PAXgene RNA tubes (Qiagen PAXgene Blood RNA IVD), reverse-transcribed into cDNA using a high-efficiency mRNA-targeted enzyme system (Lexogen QuantSeq 3’ FWD), and sequenced on an Illumina NextSeq instrument (Lexogen GmbH). Sequencing targeted 5 million reads per sample (achieved mean = 5.7 million), each of which was mapped to the GRCh38 human reference transcriptome (average 99.6% mapped) using the STAR aligner. Transcript abundance was quantified as gene transcripts per million total mapped reads (TPM), floored at 1 TPM to suppress spurious variability, log_2_ transformed to stabilize variance, and mean-centered for linear statistical model analyses as described below.

### MRI acquisition and analysis

Participants were scanned on a Philips Ingenia CX dStream 3.0 T system using a standardized acquisition protocol. This included a 3D T1-weighted sagittal MPRAGE (field of view 256 mm × 240 mm with matrix size of 256 × 240, slice thickness 1.0 mm, slice number of 181, TE = 3.2 ms, TR = 6.9 ms, TI delay = 870 ms, shot interval = 3000 ms, flip angle = 8°) and 3D FLAIR (FOV of 256 mm × 256 mm with matrix size of 256 × 256, slice thickness 1.0 mm, slice number of 181, TE = 268 ms, TR = 4800 ms, TI = 1650 ms, flip angle = 90°) sequences. MS lesions were automatically segmented from FLAIR images using the Lesion Segmentation Toolbox (v3.0)^49^ to obtain lesion counts and total T2-hyperintense lesion volume (T2LV). All T1-weighted images were processed using the automated longitudinal pipeline in the Freesurfer toolbox using the default settings^50^. Subsegmentation of the amygdala, hippocampus, brainstem and hypothalamus were performed as needed using additional Freesurfer toolboxes^51–54^.

We limited our analysis to limbic and cortical areas of interest based on our hypotheses as outlined in the introduction. Cortical surfaces were parcellated from the Destrieux atlas. The volumes (subcortical) or surface area (cortical) of the following 13 cerebral structures—by hemisphere—were exported for analysis: amygdala, hippocampus, hypothalamus, brainstem (unilateral), insula, anterior cingulate, and subcallosum. Sub-segmentation analysis of these structures was performed if significance was found at the level of the whole structure after correction for multiple comparisons.

### Statistical analysis

All variables were assessed for normality using histogram visualization and determination of skew and kurtosis, with non-normal variables undergoing either log-transformation, or non-parametric statistical tests as indicated. Descriptive statistics and pre/post assessments of psychosocial measures were determined using chi-square, Wilcoxon rank, or t-tests as appropriate. We used mixed-effects regression modeling to determine associations between MRI structural regions-of-interest (dependent variable) and patient-reported psychological measures, adjusted for age and intracranial volume as fixed effects, and subject identity as a random effect. We performed analyses both with and without the addition of prior steroid use as fixed effect. Interaction terms were introduced for specific hypothesis testing based on results. Each independent variable assessment was corrected for multiple comparisons (of the selected brain ROIs) using the Benjamini–Hochberg procedure. All aforementioned statistical analyses employed the R software (www.r-project.org).

CTRA analyses and statistics were performed separately using mixed effect linear models implemented in SAS PROC MIXED to quantify the association of study variables with average expression of 53 standard CTRA indicator gene transcripts as previously described^48^. Briefly, these analyses treated as a repeated measure the expression of 19 canonical proinflammatory response genes (*CXCL8, FOS, FOSB, FOSL1, FOSL2, IL1A, IL1B, IL6, JUN, JUNB, JUND, NFKB1, NFKB2, PTGS1, PTGS2, REL, RELA, RELB, TNF*) and 34 Type I IFN response genes (*GBP1, IFI16, IFI27, IFI27L1, IFI27L2, IFI30, IFI35, IFI44, IFI44L, IFI6, IFIH1, IFIT1, IFIT1B, IFIT2, IFIT3, IFIT5, IFITM1, IFITM2, IFITM3, IFITM4P, IFITM5, IFNB1, IGLL1, IGLL3P, IRF2, IRF7, IRF8, JCHAIN, MX1, MX2, OAS1, OAS2, OAS3, OASL*), with the latter sign-inverted to reflect their inverse contribution to the CTRA profile^48^. Among these transcripts, 7 showed minimal levels and variance in expression (SD = 0; *IFITM4P, IFITM5, IFNB1, IGLL1, IGLL3P, IL6, IL1A*) and were excluded from further analysis. Log_2_ transcript abundance values were tested for average association with study time point (pre- vs post-MBSR), while controlling for patient age, ethnicity, BMI, and two treatment variables found to empirically affect CTRA gene expression values: exposure to B cell depletion therapy, and exposure to pharmacologic glucocorticoids (with the latter quantified as prednisone-equivalent steroid dose discounted by duration since last dose). When noted, additional substantive variables such as patient-reported outcomes or MRI volumetric parameters were added to this benchmark analysis model. Models were estimated by maximum likelihood, and included a compound symmetry covariance matrix to account for correlation among residuals across transcripts (equivalent to a subject-specific random intercept).

### Consent to participate

This study was reviewed and approved by the University of Massachusetts ethics board (IRB Protocols #H00017392). Data collection, storage, and access were in accordance with the Health Insurance Portability and Accountability Act. All patients provided written informed consent prior to enrollment.

## Results

### Patient-reported measures

83% (19/23) patients completed both the baseline and follow-up questionnaires. Significant improvements were observed across nearly all of the measures, including perceived stress, anxiety, depression, fatigue, and loneliness. See Table 2 for a summary of the results.

**Table 2:** Patient reported measures pre/post MBSR.

	Pre-MBSR	Post-MBSR	
	*n* = 19^1^	*n* = 19^1^	*p*-value^2^
Brief Inventory of perceived stress			
Pushed	8.4 ± 2.9	6.9 ± 2.1	0.010
Conflict imposition	7.3 ± 2.3	5.1 ± 2.4	0.002
Lack of control	8.9 ± 2.1	6.4 ± 2.1	< 0.001
Depression, anxiety and stress scale			
Stress	9.5 ± 4.4	6.2 ± 3.5	< 0.001
Anxiety	6.2 ± 4.2	4.5 ± 4.2	0.023
Depression	6.2 ± 5.3	3.8 ± 2.8	0.009
UCLA loneliness scale	21.8 ± 7.5	17.4 ± 5.7	< 0.001
Modified fatigue inventory 5-item	3.2 ± 1.3	2.2 ± 0.9	< 0.001
Mental health continuum short form			
Hedonic well-being	3.1 ± 1.2	3.8 ± 0.8	0.002
Eudiamonic well-being	2.8 ± 1.3	3.4 ± 0.9	0.020
Psychological well-being	3.5 ± 1.1	3.9 ± 0.9	0.029

Data are presented as Mean ± SD.

^1^With complete pre/post data.

^2^Paired t-test.

### Gene expression

A sub-cohort of 12 patients provided pre- (*n* = 12) and post-MBSR (*n* = 10) blood samples starting in January 2020. All samples passed quality control metrics and were used for CTRA analysis. In a mixed-effect regression controlling for age, race, BMI, use of B-cell depletion, and recent steroid dose as fixed effects, and patient identity as a random effect, results showed no significant change in CTRA gene expression from pre- to post-MBSR (− 0.022 ± 0.058 log2 mRNA abundance, *p* = 0.715, 95% CI [− 0.156, 0.112]). However, CTRA gene expression was associated with greater loneliness (0.142 ± 0.055, *p* = 0.010, [0.034, 0.250]), and higher levels of anxiety (DASS Anxiety subscale: 0.221 ± 0.029, *p* < 0.001, [0.164, 0.277]) and stress (DASS Stress subscale: 0.231 ± 0.038, *p* < 0.001, [0.157, 0.306]; BIPS “Pushed” subscale : 0.118 ± 0.051, *p* = 0.022, [0.017, 0.218]; BIPS “Control” subscale: 0.228 ± 0.055, *p* < 0.001, [0.121, 0.336]; and BIPS conflict subscale: 0.108 ± 0.048, *p* = 0.024, [0.014, 0.202]). CTRA gene expression was inversely related to eudaimonic well-being (− 0.511 ± 0.271, *p* = 0.019, [− 0.937, − 0.086]) but not hedonic well-being (0.508 ± 0.223, *p* = 0.023, [0.070, 0.946]). Additionally, the CTRA was inversely associated with hair cortisol concentrations (− 0.093 ± 0.037, *p* = 0.011, [− 0.165, − 0.021]).

### Hair cortisol

In patients with complete paired hair sample analysis (samples = 28, *n* = 14), no significant difference in hair cortisol was detected pre- vs. post-MBSR (pre = 4.25 pg/mg; post = 3.66 pg/mg; Wilcoxon signed-rank paired test: V = 64, *p* = 0.50), after the removal of one outlying measurement in a participant using a facial product containing a steroid. The significance of this test did not change while adjusting for prior steroid use. Hair cortisol measures correlated moderately (Spearman’s ρ = 0.51, *p* = 0.002) with the total amount of exogenous glucocorticoid (prednisone equivalent) used in the prior 2.5 months. There were no differences in the amount of prior steroids received in the pre vs. post periods [pre-MBSR median = 0 mg (IQR 0, 125) and post-MBSR median = 0 mg (IQR 0, 0); Wilcoxon paired rank test: V = 38, *p*-value = 0.30].

### MRI analyses

**Figure 1:**
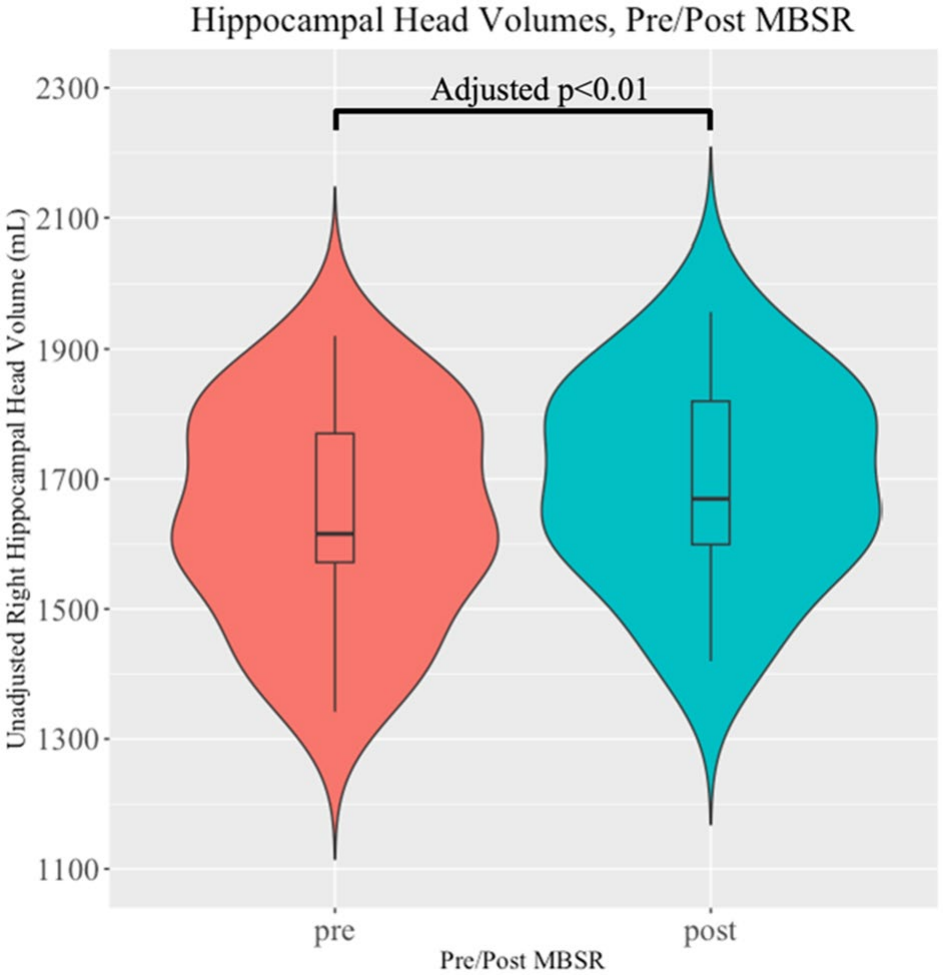
Hippocampal head volume is observed to be larger following MBSR. Violin plot showing group pre-post enlargement in right hippocampal head volume, unadjusted for covariates. The *p*-value listed is adjusted for age, intracranial volume and intra-subject correlation.

In patients who received pre- (n = 17) and post-MBSR (n = 13) MRI scans, all were of good quality and free of significant artifacts based on manual review. Table 3 summarizes the results of the 13 mixed-effects regressions with the bilateral limbic/paralimbic brain ROIs as the outcome variable and MBSR as the explanatory variable. Each regression was adjusted for age and intracranial volumes as fixed effects, and subject identity as a random effect; *p*-values were adjusted for the multiple comparisons by Benjamini–Hochberg method. Using these models we observe an association between increased right hippocampal volumes and MBSR (see Table 3). We then assessed the right hippocampus in greater detail stratified by region (head, body, tail), finding the largest association in the hippocampal head (ß = 24.2 mm^3^ larger, post-MBSR; see Fig. 1). Further subsegmentation of the hippocampal head into discrete nuclei showed significant post-MBSR enlargements in the subiculum, presubiculum, molecular layer, and CA3. These effects were attenuated but remained significant after adjusting for prior steroid use; see Table 4 for full detail. We also performed a sensitivity analysis excluding patients recently (within 3 months) started on a new or different disease-modifying therapy (N = 2), as these could potentially be associated with “pseudoatrophy”^55^. The association between MBSR and right hippocampus volume was attenuated after excluding these patients (Beta reduced from 36.6 to 32.1; unadjusted *p*-value reduced from 0.003 to 0.01; adjusted *p*-value for multiple comparisons reduced from 0.03 to 0.12). A sensitivity analysis of the symbol digit modalities test as a measure of cognitive functioning did not show any significant associations with cerebral ROIs (results not shown).

**Table 3:** MRI regions-of-interest pre/post associations with MBSR.

Dependent variable	Independent variable	Beta	LowerCI	UpperCI	*p*-value	BH-adj *p*-value
Left hippocampus	MBSR	14.0	− 19	47.1	0.421	0.632
Right hippocampus	MBSR	36.6	17.4	55.8	**0.003**	**0.033**
Left amygdala	MBSR	3.2	− 17.6	24.1	0.766	0.766
Right amygdala	MBSR	6.9	− 14.4	28.3	0.535	0.632
Left hypothalamus	MBSR	3.4	− 2.5	9.2	0.277	0.515
Right hypothalamus	MBSR	1.7	− 3.4	6.9	0.516	0.632
Left anterior cingulate	MBSR	7.8	− 4.5	20.1	0.236	0.511
Right anterior cingulate	MBSR	− 2.9	− 19.2	13.4	0.731	0.766
Left subcallosal	MBSR	− 21.3	− 51.5	8.8	0.186	0.483
Right subcallosal	MBSR	15.3	− 4.7	35.4	0.156	0.483
Left insula	MBSR	− 5.1	− 19.9	9.6	0.507	0.632
Right insula	MBSR	17.6	− 1.1	36.3	0.088	0.379
Brainstem	MBSR	122.9	0.6	245.1	0.070	0.379

Data are from a mixed-effect regression model adjusted for age, intracranial volume, and within-subject correlations as a random effect. Units for hippocampus, amygdala, brainstem and hypothalamus are mm^3^; units for cingulate, subcallosum and insula are mm^2^ (gyral cortical surface area). *BH* Benjamini–Hochberg adjustment for multiple comparisons.

Significant values are in bold.

**Table 4:** Detailed MRI assessments of the right hippocampus sub-structures associated with MBSR (pre/post).

Dependent variable	Ind. Var	ß	LowerCI	UpperCI	No steroid adjustment		Adjusted for prior steroid	
					*p*-value	BH-adj *p*-value	*p*-value	BH-adj *p*-value
R hippocampus: head	MBSR	24.2	9.9	38.6	**0.006**	**0.017**	**0.009**	**0.027**
R hippocampus: body	MBSR	11.2	0.6	21.8	0.058	0.088	0.158	0.209
R hippocampus: tail	MBSR	1.8	− 5.6	9.2	0.649	0.649	0.209	0.209
Hippocampal subsegmentation								
R subiculum: head	MBSR	2.7	0.3	5.1	**0.048**	0.087	**0.033**	0.100
R presubiculum: head	MBSR	3.7	1.1	6.3	**0.016**	**0.049**	**0.022**	0.100
R CA1: head	MBSR	4.9	− 0.4	10.2	0.092	0.138	0.163	0.245
R molecular layer HP: head	MBSR	4.4	1.6	7.2	**0.008**	**0.049**	**0.025**	0.100
R GC-ML-DG: head	MBSR	2.1	− 1	5.2	0.203	0.203	0.843	0.843
R CA4: head	MBSR	1.8	− 0.8	4.5	0.195	0.203	0.817	0.843
R CA3: head	MBSR	2.4	0.7	4.2	**0.016**	**0.049**	0.072	0.163
R HATA	MBSR	1.9	0.4	3.3	**0.027**	0.060	0.124	0.224
R parasubiculum	MBSR	0.9	− 0.3	2	0.162	0.203	0.333	0.428

*R* Right, *CA* Cornu Ammonis, *GC-ML-DG* Granule cell molecular layer of dentate gyrus, *HATA* Hippocampal-Amygdaloid Transition Area. Unit volumes are mm^3^. *BH* Benjamini–Hochberg adjustment for multiple comparisons.

Significant values are in bold.

**Figure 2:**
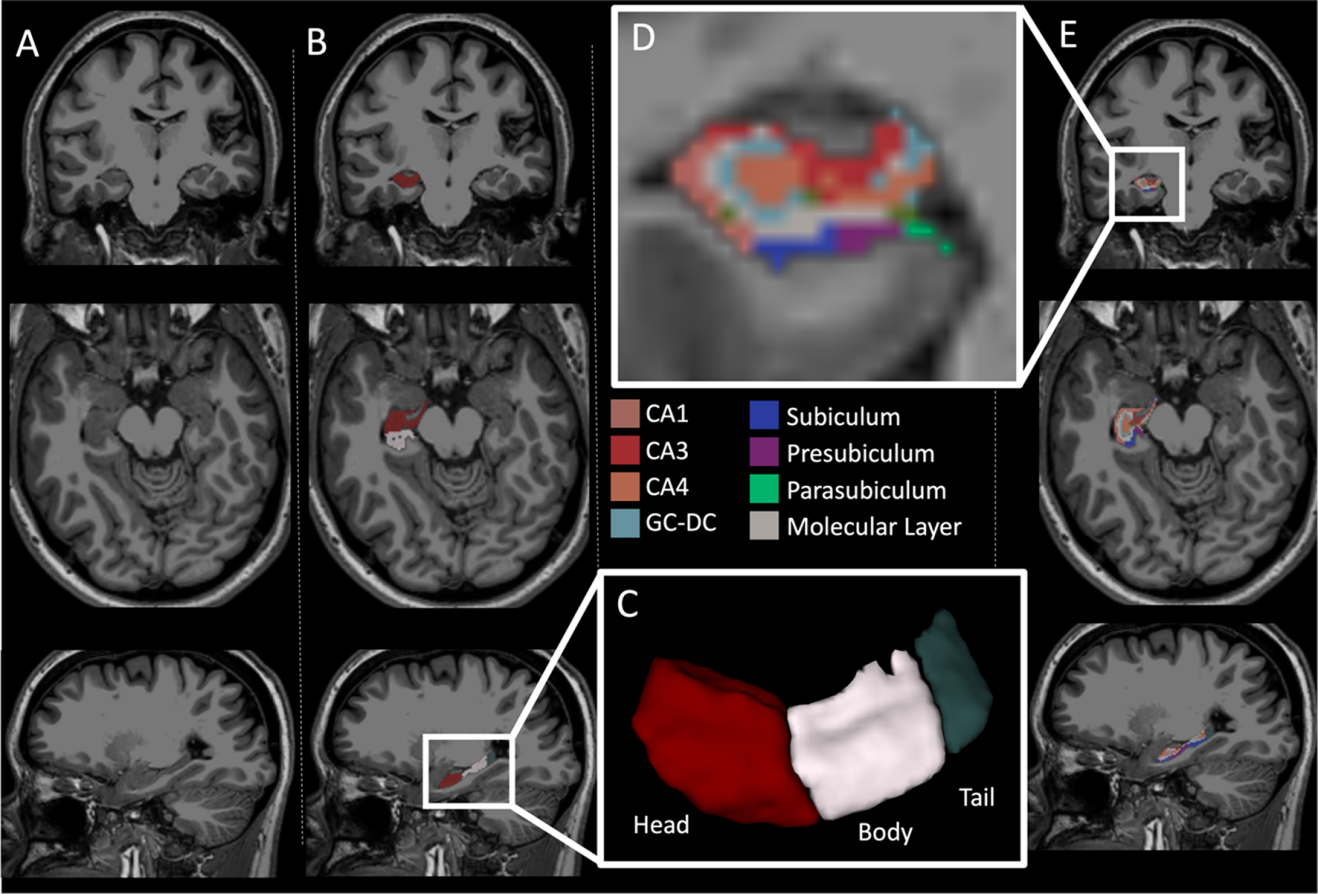
Hippocampal subsegmentation with coronal (top row), axial (middle row) and sagittal (bottom row) sections through the hippocampus. Column A is unlabeled, Column B is labeled with head/body/tail, with 3D enlargement in section C. Column E is labeled using Freesurfer hippocampal subsegmentation, with a coronal cross-section enlarged in box D.

We additionally determined associations between structural volumes of limbic/paralimbic areas and behavioral patient-reported outcomes. The full results of these analyses are presented in Supplementary Tables. In brief, few associations survived adjustment for prior steroid exposure and corrections for multiple comparisons. Notable exceptions included a negative association between right hippocampal volume and fatigue (ß = − 44.5, *p* = 0.001; *p* = 0.011 after correction for multiple comparisons and steroid use). These changes were most notable in the head of the hippocampus, in areas of the presubiculum, the subiculum, CA1, molecular layer, and CA3 (all with negative betas, all *p* < 0.05 after steroid adjustment and correction for multiple comparisons). Figure 2 shows an example of these segmented structures in one participant. There was no interaction between fatigue and MBSR (*p* > 0.05) on hippocampal volumes.

## Discussion

In this pre-post observational study of an 8-week MBSR class in pwMS, we found significant improvements in the debilitating “silent symptoms” of MS, as well as an associated enlargement of the anterior right hippocampus (head). The CTRA did not significantly change pre-post MBSR but was robustly increased with higher patient-reported levels of stress, anxiety, loneliness, and lower reported well-being in this sample. These data thus help elucidate biological mechanisms potentially underlying these symptoms in pwMS.

The leading factors affecting health-related quality of life in MS are fatigue and depression, rather than physical disability or ambulatory status^56^. Because these symptoms are not readily identified by observation (and sometimes not routinely followed in neurological practice), they are deemed “silent” or “invisible”. These challenges are compounded by a lack of pharmacological therapies proven to benefit fatigue^57^. For these reasons, any interventions that can alleviate “silent” MS symptoms are of high importance. Data from this study support a growing body of literature suggesting this low-risk educational intervention should be considered for clinical applications. Although the design of this study (lacking a control group) was not meant to demonstrate efficacy—and therefore we cannot exclude nonspecific effects—a recent meta-analysis of 14 randomized controlled trials of MSBR in MS highlights consistently high clinical value in improving quality of life^58^.

The mechanistic basis for MBSR efficacy is poorly understood. We hypothesized that MBSR would reduce both perceived stress and the CTRA through top-down modulation of sympathetic output via a convergent downstream structure, the paraventricular nucleus of the hypothalamus^37^. This master regulatory nucleus is itself regulated via a set of limbic and paralimbic areas, many of which have been previously associated with volumetric changes seen associated with MBSR such as the amygdala^27^ and hippocampus^59,60^. Here, we did observe a pre-post enlargement of right anterior hippocampal volume, most notably in the presubiculum, molecular layer and CA3; this finding was attenuated after controlling for prior exposure to steroids, which are known to affect hippocampal volumes^61^. Right hippocampal enlargement has been observed in several prior studies of mindfulness, especially in long-term meditators^59,60^. Given the small sample size this finding is at risk of being a type I error, and due to study design we cannot exclude other nonspecific effects of study participation or other uncontrolled variables in this clinical cohort. We did not find changes in any other pre-specified region that survived covariate adjustment and multiple comparisons (see Supplement for full results). In comparison to the literature, a notable recent RCT assessing structural brain changes related to MBSR (using similar acquisition and post-processing methods to this study) did not show any areas significantly different pre/post^31^, although it is possible that our group of highly motivated participants systematically differed from the healthy participants in this other study. 8 weeks may also not be a long enough duration for substantial structural changes. We also did not control for the possibility of structural disruptions related to T2-hyperintense lesion volumes in MS, although no participants had evidence of new lesions on-study.

In addition to the effects of MBSR, we observed associations between several psychosocial factors and MRI-structural volumes using mixed-effect models. Most notably, one of these associations included a negative relationship between fatigue and right hippocampal volume, specifically the anterior regions including body and head. Subsegmentation showed that greater fatigue was associated with smaller right presubiculum, subiculum, CA1, CA3, and molecular layer. This association was generally unchanged with adjustment for steroids. Results here are similar to a study of fatigue in healthy aging adults (*N* = 1374, *M*_age_ = 72 years) also using Freesurfer post-processing, that showed significant reductions in combined hippocampal volumes with greater fatigue^62^. Several studies in pwMS also showed associations between more severe fatigue and smaller bilateral^63^, or right hippocampal volumes^64^. There was no interaction between fatigue and MBSR in our data, suggesting an independent association between the two.

We observed a robust and coherent association between CTRA and measures of stress, anxiety, and loneliness, as well as protective (inverse) associations with eudaimonic well-being (but not hedonic well-being). These are novel findings in the MS population to our knowledge, and the apparently protective role of eudaimonic well-being suggests new directions for psychological interventions to improve QOL in pwMS. We did not, however, find support for our hypothesis that MBSR would be associated with changes in the CTRA. These results are in contrast with a small RCT (*N* = 40) in healthy older adults that showed reductions in NF-κB-associated gene expression following MBSR^65^. A caveat of interpretation is that much of study population (83%) were on an immunomodulatory therapy, with a substantial proportion being on B-cell depleting medications (either rituximab or ocrelizumab); this is likely reflected as the lack of IgG response in the sample, and we speculate that this could have contributed to differences in results compared to healthy populations. Another caveat is that the present study used a standard pre-specified CTRA gene composite as the outcome analyzed, whereas the previous study linking MBSR to differential gene expression used a different transcriptomic measure involving bioinformatic assessment of NF-κB gene regulation^23^. We also did not observe any neural (MRI) correlates of CTRA after adjustment for steroid use, although unadjusted analyses did reveal several structures that were larger in association with greater CTRA (these results are not presented). Exploring the structural and functional neural correlates of the CTRA remains a fruitful area for future research on bidirectional brain-immune communication; investigators should carefully control for any steroid usage or exposures among participants.

Last, we did not observe any on-study MBSR-related changes in hair cortisol as hypothesized. The reasons for this could include the small sample size, complications from steroid medication exposure, and that changes in perceived or psychological stress are not always accompanied by parallel changes in cortisol output, including output measured by cortisol accumulation in hair^66^. Hair cortisol measurements did, however, strongly reflect the use of on-study steroid exposure, supporting its validity as a biomarker.

A primary limitation of this study is the small sample size (with even smaller cohorts of complete CTRA and MRI data) that puts this study at substantial risk of type I and type II errors—a shortcoming which may lead present findings to be inconsistent with previous results. The study also focused on a population of “real world” MS patients, a dynamic neurological cohort that was biased with a 100% female demographic. These biases potentially constrain the generalizability of findings. An additional limitation of the study is the occurrence of the COVID-19 pandemic halfway through recruitment, necessitating a change in class structure from in-person to virtual and disrupting follow-up. We included all data whenever possible to maximize power, but inconsistent/missing data could also have introduced a bias. Although most demographic and clinical parameters were similar pre- and post-COVID, there was notably a significantly higher level of depression symptoms in the post-COVID cohort (see supplemental material). Notwithstanding these admonitions, we present these data and our experience as a feasible structure for future research in psycho-neuro-immunology, including the need to carefully account for potential methodological pitfalls such as steroid use and immunotherapy.

## Conclusion

MBSR remains a promising non-pharmacological strategy for addressing the debilitating “silent symptoms” of MS. The present data validate the associations between patient-reported stress, anxiety, and loneliness with greater systemic inflammation as reflected by the CTRA, and patient-reported eudiamonic well-being with reduced inflammation, in an MS population. Although we did not find any consistent associations between MBSR and the CTRA, we did observe a pre-post enlargement of right anterior hippocampal nuclei as well as negative correlations between fatigue and these same structures, findings that should be interpreted with caution due to methodological limitations. Future research directions include the assessment of CTRA changes in relation to functional brain connectivity (resting-state fMRI), which may reflect changes in the bidirectional neural-immune communication more sensitively.

### Supplementary Information

Supplementary Information.

## Data Availability

Data from this article are available to others upon reasonable request and completion of a data sharing agreement. Please contact the corresponding author (Christopher Hemond) for details.
