# Supplementary material for: A single-arm, open-label pilot study of neuroimaging, behavioral, and peripheral inflammatory correlates of mindfulness-based stress reduction in multiple sclerosis
Source: Sci Rep. 2024 Jun 18;14:14044. doi: 10.1038/s41598-024-62960-w (PMC11189512; doi:10.1038/s41598-024-62960-w)
Supplement: Supplementary file 1 — Supplementary Information. [file 41598_2024_62960_MOESM1_ESM.pdf]

Supplementary Material:

Table S1: Baseline comparison of demographic and clinical variables as stratified by in-person (pre-covid) and virtual (post-covid) class formats.

Variable	In-person, pre-COVID N = 12[1]	Virtual, post-COVID N = 10[1]	p-value[2]
Age	47.9 ± 9.7	44.0 ± 13.1	0.500
EDSS	2.2 ± 1.5	1.9 ± 0.8	0.500
Hair Cortisol	2.8 (1.8, 3.4)	5.0 (4.1, 8.3)	**0.010**
BIPS: Pushed	8.4 ± 2.2	8.8 ± 3.4	0.800
BIPS: Conflict Imposition	7.1 ± 1.9	7.8 ± 2.7	0.500
BIPS: Lack of Control	8.8 ± 2.5	9.2 ± 1.4	0.600
DASS: Stress	8.0 ± 4.3	11.4 ± 3.4	0.052
DASS: Anxiety	5.0 ± 4.4	8.3 ± 3.9	0.077
DASS: Depression	3.8 ± 4.2	9.2 ± 5.3	**0.019**
UCLA Loneliness Scale	19.8 ± 8.2	24.3 ± 6.4	0.200
MFIS Fatigue (5-item)	2.9 ± 1.3	3.7 ± 1.1	0.110
Well-being: Hedonic	3.1 ± 1.1	2.9 ± 1.4	0.700
Well-being: Eudiamonic	2.8 ± 1.4	2.6 ± 1.4	0.700
Well-being: Psychological	3.5 ± 1.2	3.3 ± 1.1	0.600
SF20: Physical	75.0 ± 35.2	69.2 ± 33.8	0.700
SF20: Role Functioning	66.7 ± 44.4	62.5 ± 46.0	0.800
SF20: Social Functioning	76.7 ± 26.7	62.0 ± 29.0	0.200
SF20: Mental Health	68.7 ± 13.3	60.0 ± 8.4	0.080
SF20: Health Perception	44.2 ± 25.4	51.0 ± 10.7	0.400
SF20: Pain	63.3 ± 29.3	44.0 ± 24.6	0.110

[1] Mean ± SD; Median (IQR); [2] Welch Two Sample t-test; Wilcoxon rank sum exact test

Table S2: Associations between patient-reported psychosocial variables and prespecified MRI regions-of-interest

		**Not adjusted for prior steroid use**					**Adjusted for prior steroid use**				
Dependent Variable	Independent Variable	Beta	LowerCI	UpperCI	PValue	AdjPValue	Beta	LowerCI	UpperCI	PValue	AdjPValue
Left Hippocampus	BIPS_pushed	-17.1	-29.5	-4.7	**0.016**	0.104	-26.2	-41.1	-11.4	**0.003**	**0.042**
Right Hippocampus	BIPS_pushed	-14.1	-24.2	-4	**0.016**	0.104	-9.7	-22.5	3.1	0.163	0.364
Left Amygdala	BIPS_pushed	-1.6	-10.2	7.1	0.727	0.788	-1.6	-12	8.8	0.769	0.769
Right Amygdala	BIPS_pushed	-9.7	-17.6	-1.8	**0.03**	0.129	-7.8	-18.2	2.7	0.168	0.364
Left Hypothalamus	BIPS_pushed	-1.3	-3.8	1.2	0.306	0.497	-1.3	-4.5	1.8	0.41	0.667
Right Hypothalamus	BIPS_pushed	-1.2	-3.5	1	0.298	0.497	-0.3	-2.6	1.9	0.766	0.769
Left Anterior Cingulate	BIPS_pushed	2.1	-3.8	8.1	0.498	0.648	2.4	-6.2	11	0.59	0.767
Right Anterior Cingulate	BIPS_pushed	4.6	-2.6	11.8	0.229	0.496	11.6	3.9	19.2	**0.012**	0.079
Left Subcallosal	BIPS_pushed	5.9	-2.5	14.4	0.179	0.466	6.9	-1.3	15	0.112	0.364
Right Subcallosal	BIPS_pushed	5.8	-1.4	12.9	0.126	0.408	8.7	1.8	15.7	**0.022**	0.096
Left Insula	BIPS_pushed	1.5	-5.5	8.4	0.682	0.788	-1.9	-6.9	3	0.464	0.67
Right Insula	BIPS_pushed	-3.4	-12.9	6.1	0.493	0.648	-5.8	-18.4	6.7	0.38	0.667
Brainstem	BIPS_pushed	-7.1	-70.1	55.9	0.829	0.829	17.4	-67.7	102.5	0.696	0.769
Left Hippocampus	BIPS_conflict-imposition	-1.9	-12.8	8.9	0.732	0.805	-3.4	-17.3	10.5	0.637	0.756
Right Hippocampus	BIPS_conflict-imposition	-9.1	-16.8	-1.5	**0.035**	0.454	-5.6	-14.5	3.3	0.241	0.756
Left Amygdala	BIPS_conflict-imposition	1.1	-5.5	7.7	0.744	0.805	0.2	-7.8	8.2	0.966	0.966
Right Amygdala	BIPS_conflict-imposition	-4.2	-10.9	2.5	0.238	0.595	-2.1	-10.2	6	0.623	0.756
Left Hypothalamus	BIPS_conflict-imposition	-0.2	-2.2	1.7	0.805	0.805	0.6	-1.9	3.1	0.64	0.756
Right Hypothalamus	BIPS_conflict-imposition	-0.5	-2.1	1.2	0.576	0.805	-0.1	-1.6	1.5	0.95	0.966
Left Anterior Cingulate	BIPS_conflict-imposition	1.7	-2.5	6	0.444	0.797	1.9	-3.7	7.6	0.516	0.756
Right Anterior Cingulate	BIPS_conflict-imposition	0.9	-4.5	6.4	0.74	0.805	3.1	-3.3	9.6	0.359	0.756
Left Subcallosal	BIPS_conflict-imposition	-5.7	-13.3	1.9	0.154	0.595	-4.5	-12.7	3.8	0.298	0.756
Right Subcallosal	BIPS_conflict-imposition	-3.5	-9.5	2.6	0.275	0.595	-2.3	-9.5	4.8	0.531	0.756
Left Insula	BIPS_conflict-imposition	-1.8	-6.7	3.1	0.491	0.797	-1.5	-4.9	1.8	0.38	0.756
Right Insula	BIPS_conflict-imposition	-6	-12.2	0.3	0.084	0.543	-8	-15.7	-0.4	0.063	0.756
Brainstem	BIPS_conflict-imposition	-29.7	-71.7	12.4	0.19	0.595	-22.2	-77.6	33.2	0.449	0.756
Left Hippocampus	BIPS_lack-control	-7.8	-18.5	2.9	0.176	0.515	-12.2	-26.4	2	0.117	0.492
Right Hippocampus	BIPS_lack-control	-13.2	-19.4	-7.1	**0.001**	**0.012**	-10.9	-18.8	-2.9	**0.021**	0.162
Left Amygdala	BIPS_lack-control	-0.2	-7.1	6.7	0.96	0.96	0.2	-8.5	8.8	0.97	0.97
Right Amygdala	BIPS_lack-control	-3.7	-10.6	3.2	0.307	0.57	0.2	-8.4	8.9	0.959	0.97
Left Hypothalamus	BIPS_lack-control	-1.2	-3.1	0.7	0.225	0.515	-1.5	-3.9	1	0.258	0.612
Right Hypothalamus	BIPS_lack-control	-1	-2.7	0.6	0.238	0.515	-0.3	-1.9	1.3	0.726	0.944
Left Anterior Cingulate	BIPS_lack-control	-0.4	-4.8	4.1	0.872	0.945	-1.3	-7.3	4.7	0.677	0.944
Right Anterior Cingulate	BIPS_lack-control	2.3	-3.2	7.7	0.433	0.704	5.1	-1.4	11.7	0.151	0.492
Left Subcallosal	BIPS_lack-control	0.9	-7.9	9.6	0.85	0.945	3.8	-5.7	13.4	0.438	0.814
Right Subcallosal	BIPS_lack-control	-1.2	-7.8	5.4	0.729	0.945	1.7	-6.4	9.8	0.682	0.944
Left Insula	BIPS_lack-control	0.7	-4.4	5.8	0.796	0.945	0.2	-3.4	3.9	0.898	0.97
Right Insula	BIPS_lack-control	-6.1	-12.6	0.5	0.092	0.515	-10.1	-17.8	-2.4	**0.025**	0.162

**Brainstem**	BIPS_lack-control	-35	-77.7	7.6	0.131	0.515	-33.2	-90.7	24.4	0.282	0.612
		**Not adjusted for prior steroid use**					**Adjusted for prior steroid use**				
**DependentVar**	IndependentVar	Beta	LowerCI	UpperCI	PValue	AdjPValue	Beta	LowerCI	UpperCI	PValue	AdjPValue
Left Hippocampus	DASS_stress	-3.6	-10.8	3.7	0.353	0.684	-4.5	-13	4.1	0.324	0.584
Right Hippocampus	DASS_stress	-4.6	-10.4	1.1	0.139	0.618	-4.6	-10	0.7	0.116	0.501
Left Amygdala	DASS_stress	3.6	-1	8.2	0.148	0.618	5.6	0.2	10.9	0.057	0.369
Right Amygdala	DASS_stress	1.7	-3.1	6.4	0.501	0.684	2.7	-2.4	7.7	0.32	0.584
Left Hypothalamus	DASS_stress	-0.4	-1.7	0.9	0.526	0.684	0	-1.5	1.5	0.961	0.961
Right Hypothalamus	DASS_stress	0.4	-0.7	1.6	0.45	0.684	0.2	-0.8	1.1	0.716	0.924
Left Anterior Cingulate	DASS_stress	0.7	-2.3	3.6	0.653	0.772	0.4	-3.3	4	0.853	0.924
Right Anterior Cingulate	DASS_stress	0.6	-3.2	4.3	0.774	0.774	2.5	-1.6	6.6	0.255	0.584
Left Subcallosal	DASS_stress	2.1	-2.8	7.1	0.406	0.684	3.3	-1.6	8.3	0.195	0.584
Right Subcallosal	DASS_stress	-0.7	-4.7	3.3	0.734	0.774	0.6	-3.8	4.9	0.803	0.924
Left Insula	DASS_stress	2.3	-0.9	5.5	0.19	0.618	1	-1.1	3.1	0.36	0.584
Right Insula	DASS_stress	-2.2	-6.8	2.4	0.369	0.684	-2	-7.5	3.4	0.478	0.691
Brainstem	DASS_stress	-28.2	-55.3	-1.2	0.061	0.618	-37.8	-66.4	-9.2	**0.024**	0.316
Left Hippocampus	DASS_anxiety	0.6	-8.3	9.5	0.898	0.957	-0.8	-11.6	10	0.882	0.99
Right Hippocampus	DASS_anxiety	-4.8	-12	2.5	0.217	0.765	-4.4	-11.6	2.8	0.258	0.99
Left Amygdala	DASS_anxiety	7.2	2.1	12.4	**0.01**	0.135	7.2	1.2	13.2	**0.027**	0.355
Right Amygdala	DASS_anxiety	0.2	-5.5	6	0.939	0.957	0.2	-6.1	6.6	0.945	0.99
Left Hypothalamus	DASS_anxiety	-0.1	-1.6	1.4	0.893	0.957	0.4	-1.4	2.2	0.644	0.99
Right Hypothalamus	DASS_anxiety	-0.6	-2	0.7	0.383	0.957	-0.8	-1.9	0.3	0.178	0.99
Left Anterior Cingulate	DASS_anxiety	-0.1	-3.8	3.6	0.957	0.957	-0.1	-4.8	4.5	0.955	0.99
Right Anterior Cingulate	DASS_anxiety	0.5	-4.1	5.2	0.83	0.957	0.9	-4.5	6.4	0.744	0.99
Left Subcallosal	DASS_anxiety	-0.2	-5.1	4.7	0.926	0.957	1.6	-3.7	6.8	0.558	0.99
Right Subcallosal	DASS_anxiety	0.2	-4	4.4	0.918	0.957	1.6	-3	6.3	0.497	0.99
Left Insula	DASS_anxiety	-2.9	-6.8	1.1	0.176	0.765	0	-2.8	2.8	0.99	0.99
Right Insula	DASS_anxiety	-3.5	-9.1	2.1	0.235	0.765	-3.2	-10.1	3.6	0.374	0.99
Brainstem	DASS_anxiety	-11.7	-49.5	26.2	0.555	0.957	-3.5	-49.8	42.7	0.883	0.99
Left Hippocampus	DASS_depression	-0.7	-8.3	6.8	0.853	0.971	-0.5	-9	8.1	0.917	0.992
Right Hippocampus	DASS_depression	-6.4	-11.8	-1	**0.035**	0.266	-5.9	-10.6	-1.2	**0.03**	0.195
Left Amygdala	DASS_depression	0.1	-4.4	4.6	0.971	0.971	0.7	-4.2	5.6	0.774	0.992
Right Amygdala	DASS_depression	-1.2	-6	3.6	0.64	0.971	-0.4	-5.3	4.5	0.883	0.992
Left Hypothalamus	DASS_depression	0.4	-0.9	1.7	0.553	0.971	0.7	-0.7	2.2	0.334	0.992
Right Hypothalamus	DASS_depression	0.2	-1	1.4	0.734	0.971	0	-0.9	0.9	0.992	0.992
Left Anterior Cingulate	DASS_depression	-0.7	-3.7	2.4	0.679	0.971	-1.2	-4.6	2.3	0.522	0.992
Right Anterior Cingulate	DASS_depression	-0.4	-4.2	3.5	0.858	0.971	0.7	-3.4	4.8	0.75	0.992
Left Subcallosal	DASS_depression	0.2	-4.7	5	0.949	0.971	1.1	-3.8	6	0.667	0.992
Right Subcallosal	DASS_depression	-0.3	-4.2	3.6	0.882	0.971	0.3	-3.9	4.5	0.891	0.992
Left Insula	DASS_depression	2.2	-1.1	5.4	0.22	0.952	0.9	-1.2	2.9	0.428	0.992
Right Insula	DASS_depression	-2.4	-7.1	2.3	0.334	0.971	-2.7	-7.9	2.5	0.335	0.992

**Brainstem**	DASS_depression	-31	-57.9	-4.1	**0.041**	0.266	-36.1	-63.9	-8.2	**0.027**	0.195

Table S3: Associations between patient-reported Loneliness (top) and Fatigue (bottom) with MRI regions-of-interest

		**Not adjusted for prior steroid use**					Adjusted for prior steroid use				
Dependent Variable	Independent Variable	Beta	LowerCI	UpperCI	PValue	AdjPValue	Beta	LowerCI	UpperCI	PValue	AdjPValue
Left Hippocampus	Loneliness_avg	-1.1	-6.1	3.8	0.655	0.774	-1.2	-7	4.5	0.676	0.736
Right Hippocampus	Loneliness_avg	-3.3	-7	0.5	0.115	0.72	-3.6	-6.9	-0.3	0.058	0.374
Left Amygdala	Loneliness_avg	1.3	-1.8	4.3	0.438	0.758	1.9	-1.6	5.3	0.312	0.677
Right Amygdala	Loneliness_avg	1.1	-2.2	4.3	0.526	0.758	1.6	-1.8	5	0.383	0.677
Left Hypothalamus	Loneliness_avg	0	-0.9	0.9	0.981	0.981	0.4	-0.6	1.5	0.416	0.677
Right Hypothalamus	Loneliness_avg	0.3	-0.5	1.1	0.482	0.758	-0.1	-0.7	0.5	0.736	0.736
Left Anterior Cingulate	Loneliness_avg	0.9	-1.1	2.8	0.402	0.758	0.7	-1.6	3.1	0.552	0.718
Right Anterior Cingulate	Loneliness_avg	-0.1	-2.6	2.4	0.934	0.981	0.9	-1.9	3.7	0.525	0.718
Left Subcallosal	Loneliness_avg	0.9	-2.3	4.2	0.583	0.758	1.5	-1.8	4.8	0.372	0.677
Right Subcallosal	Loneliness_avg	-1	-3.6	1.7	0.476	0.758	-0.6	-3.4	2.2	0.685	0.736
Left Insula	Loneliness_avg	1.5	-0.7	3.7	0.2	0.72	1	-0.4	2.3	0.178	0.677
Right Insula	Loneliness_avg	-2	-5.1	1.1	0.222	0.72	-2	-5.6	1.6	0.301	0.677
Brainstem	Loneliness_avg	-20.9	-38.4	-3.5	**0.035**	0.454	-27.3	-44.9	-9.7	**0.011**	0.141
Left Hippocampus	MFIS-5_avg	-13.1	-55	28.8	0.548	0.792	-11.9	-47	23.1	0.514	0.831
Right Hippocampus	MFIS-5_avg	-40	-59.5	-20.5	**0.002**	**0.024**	-44.5	-65.1	-23.9	**0.001**	**0.011**
Left Amygdala	MFIS-5_avg	4.2	-19.6	27.9	0.733	0.937	1.4	-19.6	22.3	0.898	0.955
Right Amygdala	MFIS-5_avg	-12.4	-36.1	11.4	0.324	0.703	-17.6	-38.7	3.6	0.123	0.546
Left Hypothalamus	MFIS-5_avg	-0.6	-7.8	6.5	0.865	0.937	-1.5	-7.7	4.7	0.632	0.831
Right Hypothalamus	MFIS-5_avg	0.4	-4.3	5.2	0.861	0.937	-0.2	-5.8	5.5	0.955	0.955
Left Anterior Cingulate	MFIS-5_avg	-14.2	-30.1	1.8	0.109	0.473	-8.7	-22.7	5.2	0.241	0.627
Right Anterior Cingulate	MFIS-5_avg	12.2	-8.2	32.5	0.266	0.691	4.5	-14	23	0.639	0.831
Left Subcallosal	MFIS-5_avg	8.8	-12.3	30	0.421	0.767	1.4	-18.7	21.5	0.893	0.955
Right Subcallosal	MFIS-5_avg	-0.1	-19	18.8	0.992	0.992	-4.9	-21.9	12.1	0.577	0.831
Left Insula	MFIS-5_avg	4.1	-6.6	14.7	0.472	0.767	8.9	-7.5	25.3	0.306	0.663
Right Insula	MFIS-5_avg	-22.5	-47.5	2.5	0.102	0.473	-17.5	-39.4	4.5	0.141	0.546
Brainstem	MFIS-5_avg	-111.7	-282.3	58.8	0.223	0.691	-107.6	-252.7	37.6	0.168	0.546

Table S4: Associations between patient-reported fatigue and volume measures of the right hippocampus anatomical sub-regions

		Not adjusted for prior steroid use					Adjusted for prior steroid use				
Dependent Variable	Ind. Var	ß	LowerCI	UpperCI	P-Value	BH-adj P-Value	ß	LowerCI	UpperCI	P-Value	BH-adj P-Value
R Hippocampus: Head	MFIS-5_avg	-30.74	-44.80	-16.67	**0.00**	**0.00**	-21.93	-31.25	-12.61	**0.00**	**0.00**
R Hippocampus: Body	MFIS-5_avg	-15.01	-26.40	-3.62	**0.02**	**0.03**	-14.92	-27.03	-2.81	**0.03**	**0.05**
R Hippocampus: Tail	MFIS-5_avg	-0.86	-8.90	7.19	0.84	0.84	-5.30	-13.70	3.10	0.24	0.24
Hippocampal Subsegmentation:											
R Subiculum: Head	MFIS-5_avg	-2.78	-5.45	-0.12	0.06	0.09	-3.74	-6.69	-0.79	**0.03**	**0.05**
R Presubiculum: Head	MFIS-5_avg	-3.84	-6.45	-1.24	**0.01**	**0.02**	-4.54	-7.55	-1.54	**0.01**	**0.03**
R CA1: Head	MFIS-5_avg	-7.17	-12.55	-1.79	**0.02**	**0.05**	-4.85	-8.01	-1.68	**0.01**	**0.03**
R molecular layer HP: Head	MFIS-5_avg	-5.79	-8.43	-3.15	**0.00**	**0.01**	-4.84	-7.13	-2.56	**0.00**	**0.01**
R GC-ML-DG: Head	MFIS-5_avg	-2.88	-5.85	0.09	0.07	0.09	-0.74	-2.82	1.34	0.50	0.56
R CA4: Head	MFIS-5_avg	-2.52	-5.05	0.01	0.07	0.09	-0.83	-3.01	1.34	0.47	0.56
R CA3: Head	MFIS-5_avg	-3.06	-4.80	-1.32	**0.00**	**0.02**	-1.84	-3.21	-0.47	**0.02**	**0.05**
R HATA	MFIS-5_avg	-1.31	-2.97	0.34	0.14	0.14	-0.41	-1.82	1.00	0.58	0.58
R Parasubiculum	MFIS-5_avg	-1.09	-2.40	0.23	0.13	0.14	-1.04	-2.62	0.53	0.22	0.32
